# Prevalence and mapping of hepatitis C infections among men who have sex with men in New York City

**DOI:** 10.1371/journal.pone.0200269

**Published:** 2018-07-18

**Authors:** Hong-Van Tieu, Oliver Laeyendecker, Vijay Nandi, Rebecca Rose, Reinaldo Fernandez, Briana Lynch, Donald R. Hoover, Victoria Frye, Beryl A. Koblin

**Affiliations:** 1 Laboratory of Infectious Disease Prevention, New York Blood Center, New York, United States of America; 2 Columbia University Medical Center, Division of Infectious Diseases, Department of Medicine, New York, United States of America; 3 National Institute of Allergy and Infectious Diseases, Baltimore, United States of America; 4 School of Medicine, Johns Hopkins University, Baltimore, United States of America; 5 Laboratory of Data Analytics, New York Blood Center, New York, United States of America; 6 BioInfoExperts, LLC, Thibodaux, United States of America; 7 Rutgers the State University of New Jersey, Department of Statistics, Piscataway, United States of America; 8 City University of New York School of Medicine, New York, United States of America; Centers for Disease Control and Prevention, UNITED STATES

## Abstract

Emerging sexually transmitted hepatitis C virus (HCV) epidemics among men who have sex with men (MSM) have been reported worldwide, with higher HCV infection rates among those who are HIV-infected. This study aims to determine prevalence of recent and chronic HCV infections among community-recruited MSM in New York City (NYC), map HCV infections by home, social, and sexual neighborhoods, and identify clusters of genetically linked HCV variants using phylogenetic analysis. The NYC M2M study recruited MSM via modified time-space, venue-based sampling and internet/mobile app-based recruitment during 2010–13. Participants completed a Google Earth map on neighborhoods of where they lived, socialized, and had sex in the last 3 months, an ACASI questionnaire, and a sexual network inventory about their sex partners. The men received HIV testing and provided serum samples. Testing on stored serum samples included HCV antibody and RNA viral load, HCV antibody avidity assay (avidity index <30% with positive viral load is considered recently infected), and HCV RNA extraction and amplification to generate a 432 base-pair region of Core/E1 for sequencing and phylogenetic analysis. Historic local controls were included in the phylogenetic analysis. Of 1,028 MSM, 79.7% were HIV-negative and 20.3% HIV-positive. Twenty nine MSM (2.8%) were HCV antibody-positive. MSM who were HCV antibody-positive reported a median of 2 male sex partners in last 3 months, with 6.9% aged 18–24, 17.2% 25–29, 13.8% 30–39, and 62.1% 40 and over. 8.1% of HIV-positive MSM were HCV antibody-positive vs. 1.5% of HIV-negative men (p<0.0001). Of 29 HCV-antibody positive MSM, 12 (41%) were HCV RNA-positive (11 subtype 1a and 1 subtype 1b). Two of 12 HCV RNA-positive participants had low antibody avidity values, suggesting recent HCV infection. HCV antibody seropositivity was significantly associated with older age >40 years, adjusted odds ratio (aOR) 3.56 (95% CI 1.57, 8.08), HIV-positive serostatus, aOR 3.18 (95% CI 1.40, 7.22), any sexually transmitted infection (STI) in the last 3 months, aOR 2.81 (95% CI 1.11, 7.13), and injection drug use (IDU) ever, aOR 4.34 (95% CI 1.69, 11.17). Mapping of HCV infections differed slightly by home, social, and sexual neighborhoods. Based on phylogenetic analysis from 12 HCV RNA-positive samples, no evidence of a clustered HCV epidemic was found. Overall HCV seroprevalence was 2.8% among community-recruited MSM in NYC, with higher prevalence among HIV-positive MSM compared to HIV-negative MSM. Only two participants were found to have recent HCV infection, with no evidence of a clustered HCV epidemic based on phylogenetic analysis. Our results support testing of HCV infection among HIV-negative MSM if they report having a recent STI and IDU in the past rather than universal HCV testing in all HIV-negative MSM.

## Introduction

Although primary transmission of hepatitis C virus (HCV) is through the parenteral route[1], epidemics of sexually transmitted HCV infection among men who have sex with men (MSM) have been reported in Europe, Australia, Asia, and North America, with blood remaining the likely medium for sexual transmission of HCV in these outbreaks[2–14]. These sexually transmitted HCV outbreaks have mostly involved HIV-infected MSM, with lower HCV infection rates in HIV-negative MSM[13, 15–26]. Many studies have also reported strong associations between HCV infection risk and sexual risk behaviors, including high number of sexual partners[10, 27], condomless anal sex[10, 11, 17, 27], recreational drug use during sex[10, 11], and traumatic sexual practices such as fisting[10, 27, 28]. In the Multicenter AIDS Cohort Study (MACS) study of HCV infections among HIV positive and HIV negative MSM in four U.S. metropolitan areas from 1984–2011, 115 cases of incident HCV infection were reported (incidence rate of 2.08/1,000 person-years [PYs]). In multivariate analysis, condomless receptive anal sex with more than one male partner, older age, HIV infection, heavy alcohol use, hepatitis B surface antigen positivity, and syphilis infection were significantly associated with incident HCV infection[17]. High rates of HCV reinfection of up to 15/100 PYs have been reported among HIV-infected MSM who had previously received successful HCV treatment or had spontaneously cleared their HCV infections[29–32].

Untreated HCV infection leads to chronic hepatitis in 60–80% of cases. Spontaneous clearance of HCV without treatment is seen in approximately 25% of exposed individuals in the first 6 months. Serious complications of chronic hepatitis include liver cirrhosis and hepatocellular carcinoma[1, 33–37]. With the introduction of new, oral direct acting antiviral drugs, treatment success rates for chronic HCV infection have improved markedly[38–40]. Interferon-based HCV treatment early after diagnosis is associated with higher rates of sustained virologic response compared to delayed treatment of chronic HCV infection[41–44], with the use of direct acting antiviral drugs under investigation for recent HCV infection. Early treatment has public health implications by potentially decreasing the risk of onward sexual and parenteral transmission of HCV to partners. Cost-effectiveness studies show that early HCV treatment after diagnosis maximizes medical costs savings and gains in quality-adjusted life years[45–47].

Given the benefits of early compared to late HCV treatment, accurate diagnosis of recent HCV infection is important. Clinical diagnosis of recent HCV infection can be challenging since newly infected individuals are frequently asymptomatic[48]. Diagnosis can also be delayed and problematic, as it can be difficult to distinguish between acute, resolved, and chronic infections when using immunoglobulin M (IgM) anti-HCV testing[48, 49].HCV antibody avidity assays are research tools that measure the binding strength of anti-HCV antibodies in infected individuals. These assays can be used in research to distinguish recent infection from chronic and resolved HCV infections[48, 50–56]. HCV antibody avidity increases gradually over the course of HCV infection: antibodies produced early in infection have weak antigen-binding ability while mature antibodies generated later in infection have strong antigen-binding capacity. HCV antibody avidity assays can provide advantages over traditional laboratory diagnosis of recent HCV infection and can be important for estimating HCV incidence using cross-sectional research data[55].

This study aims to determine prevalence of recent and chronic HCV infections among community-recruited MSM in New York City (NYC) by using HCV antibody avidity assays, determine sociodemographic and risk behavior characteristics that are associated with HCV infection, map HCV infections by home, social, and sexual neighborhoods, and identify clusters of genetically linked HCV variants using phylogenetic analysis. This study is based on the NYC M2M study (NIH R01 HD059729, PI: Koblin), a cross-sectional study conducted between 2010–2013 to identify urban environment characteristics that influence sexual risk behaviors, substance use, and depression among 1,458 MSM in NYC[57–61].

## Materials and methods

The NYC M2M study was approved by the Institutional Review Board (IRB) of the New York Blood Center, and this identity unlinked substudy was also approved by the New York Blood Center IRB. No new samples or behavioral data were acquired for this substudy. All laboratory testing was performed in a research laboratory incapable of releasing results to patients or clinicians. All participants in the NYC M2M study were contacted by study staff upon completion of laboratory analyses with a summary of study findings and referral to various clinics in NYC for clinical HCV testing and medical management as needed.

As described in detail previously[58, 59], MSM from the main NYC M2M study were recruited in person using a modified venue-based, time-space sampling methodology and internet- and mobile application-based recruitment. They were eligible for the study if they self-reported biological male sex at birth; engaged in anal sex with a man in the past 3 months; at least 18 years of age; resided in New York City; communicated in English or Spanish; and were willing and able to give informed consent for the study. Interested participants were referred to the study website and screened for preliminary eligibility. Study staff made attempts to contact preliminarily eligible participants to further screen for eligibility and schedule an on-site study visit.

After providing written informed consent, participants completed the Neighborhood Locator Questionnaire (NLQ) with study staff. This questionnaire collected information on the location of 4 neighborhoods: home (where they lived), social (where they socialized most often) and sexual (where they most recently had sex and where they most often had sex in the last 3 months). The men identified the neighborhoods by utilizing Google Earth on a computer to ‘drop a pin’ at the closest intersection. Geospatial coordinates (latitude and longitude) of the pin drop for each type of neighborhood were recorded. Next, the participants completed an Audio Computer-Assisted Self-Interview Software (ACASI) questionnaire. Next, a social and sexual network questionnaire (SSNQ) was completed with an interviewer with data entry into a computer system. Participants were asked, using a name generator, to name up to 10 people with whom they have had a social relationship and up to 15 sexual partners with whom they have had anal or vaginal sex in the last 3 months.

Participants then received HIV risk reduction counseling and were offered a rapid HIV antibody test (Orasure), with reactive HIV tests confirmed by Western Blot. Participants with HIV infection (newly diagnosed and previously known infection) were asked to provide a blood sample to test for CD4 cell count and HIV viral load. A total of 10 mL blood sample was collected from the participants. Participants testing HIV-positive were referred for treatment and services. Participants who consented to blood storage had remaining serum samples aliquoted and stored frozen at -80°C. Participants were reimbursed $50 and a Metrocard for their time and transportation cost.

### Laboratory analysis of serum samples

#### HCV antibody

HCV antibody testing was performed using the Genedia HCV ELISA 3.0 (Green Cross Medical Science, Korea) as per manufacturer’s protocol. Confirmatory Recombinant ImmunoBlot Assay (RIBA) testing was not performed.

#### HCV RNA viral load

HCV RNA viral load testing was performed using the Abbott Real Time HCV Amplification Reagent Kit (No. 04J86-90, Des Plaines, Illinois), with a limit of detection of 50 IU/mL.

#### HCV antibody avidity assay

The identification of individuals with recent infection was based on a HCV antibody avidity assay using diethylamine (DEA) as a chaotropic agent[55]. An avidity index was calculated by determining the percentage that the DEA reduces in optical density of the sample in comparison to a paired non-treated well. An avidity index <30% was considered recently infected. The algorithm for defining recent HCV infection was an avidity index <30% and a positive HCV RNA viral load. We used a method where individuals appeared recently infected for a mean of 147 days after seroconversion and < 1% of 579 chronically infected individuals (>2 years post infection) were misclassified[55]. The false recent rate used was 1.1%, properly adjusted for the HIV prevalence of the cohort[55].

#### HCV phylogenetics

HCV ribonucleic acid (RNA) was extracted and amplified to generate a 432 base-pair region of Core/E1 for sequencing. HCV genotype was determined. All sequence data generated from participants, reference sequences for subtype 1a and 1b, and unrelated sequences from NYC study of people who inject drugs[62] were used to generate a maximum likelihood tree using PhyML with 200 bootstraps. Phylogenies were estimated using a maximum likelihood approach and those sequences linked by nodes with bootstrap support >70 considered clustered.

### Measures

#### ACASI

Data on age, race/ethnicity, education, household income, and self-reported HIV serostatus were collected via the ACASI questionnaire. Alcohol and drug use in the prior 3 months was also collected, including use of marijuana, inhaled nitrates, smoked and powder cocaine, methamphetamine, heroin, non-prescribed hydrocodone bitartrate and acetaminophen/oxycodone/alprazolam, sildenafil/tadalafil /vardenafil, hallucinogens, injection drug use (IDU), anabolic steroids and female hormones. In addition, IDU ever in the past was asked. Self-reported history of sexually transmitted infections (STIs) in the last 3 months included any genital or rectal gonorrhea or chlamydia infections, any genital or rectal herpes infection, any syphilis infection, and any genital or rectal sores or discharge.

#### Network questionnaire

For the SSNQ, each participant was asked to name up to 10 persons whom he could rely on for functional support using four domains. In addition, participants were asked about their partners with whom they had anal or vaginal sex in the last 3 months using a name generator with up to 15 sex partners. Detailed questions were asked about each named sexual partner, including socio-demographics, partner type, where participant met the partner, frequency of anal or vaginal sex and condom use with sex with the partner in the last 3 months, and specific drug or alcohol use during anal sex with or without condom use in the last 3 months with the partner.

### Statistical methods

The participant’s HIV status was determined by a combination of the self-reported HIV status on ACASI questionnaire and HIV test result (positive, negative, indeterminate) at the site. If a participant self-reported being HIV-positive but deferred or refused an HIV test at the study visit, he was categorized as HIV-positive. If a participant self-reported being HIV-negative or unknown status but tested HIV-positive on the HIV test, he was categorized as HIV-positive for this analysis.

HCV infections based on laboratory testing on stored serum samples of NYC M2M participants were classified as follows: (1) recent HCV infection (HCV antibody and RNA positive, and recent infection by HCV antibody avidity assay), (2) chronic HCV infection (HCV antibody and RNA positive, no recent infection by HCV antibody avidity assay), (3) resolved HCV infection with clearance (HCV antibody positive, RNA negative; either those who were successfully treated for HCV infection or had spontaneously cleared HCV), and (5) no HCV exposure. Prevalence of HCV antibody seropositivity (recent, chronic, and resolved HCV infections) was computed and stratified by HIV serostatus (positive, negative/indeterminate) based on testing and self-report at the study visit; 95% confidence intervals (CI) were calculated.

To determine factors that were independently associated with HCV antibody seropositivity (recent, chronic, and resolved HCV infections) among MSM in NYC, the following variables were included: sociodemographics (age, race/ethnicity, education, annual household income, employment), HIV serostatus, history of IDU in last 3 months and ever in the past, general alcohol use, IDU and non-IDU [from ACASI], self-reported STIs [from ACASI], alcohol and substance use during condomless sex [from SSNQ], and sexual risk behaviors such as number of male, transgender, and female partners, having an anonymous sex partner, having a sex partner met on the internet or mobile application, any condomless insertive anal intercourse with a male partner, and any condomless receptive anal intercourse [all from SSNQ]. Differences in HCV seropositivity were compared by sociodemographic, HIV serostatus, and risk behavior variables using Chi-square or Fisher’s exact tests. Characteristics that were significant with p-value ≤0.10 in the Chi-square or Fisher’s exact tests were included in multivariable logistic regression models to determine factors associated with having HCV seropositivity. A forward selection process was used to identify variables to include in the final multivariable models. A two-sided p-value ≤0.05 was considered statistically significant. All data analyses were conducted in SAS version 9.3 (SAS Institute Inc., Cary, NC, USA).

To map HCV antibody seropositivity (recent, chronic, and resolved HCV infections) by home, social, and sexual neighborhoods among MSM in NYC, the 4 neighborhoods (home, social, sexual with most frequent sex and last sex) collected from the NLQ of each participant with HCV infection was assigned to a neighborhood tabulation unit (NTA), which are census tract aggregations to the level of neighborhood or multiple neighborhoods currently used by the NYC Department of City Planning. Study data were imported into ArcGIS (ESRI 2012. ArcGIS Desktop: Release 10.1. Redlands, CA: Environmental Systems Research Institute) and used to map the HCV seroprevalence data. ArcGIS shapefiles for the NYC community district boundaries, water and non-residential areas were downloaded from NYC BYTES of the BIG APPLE™ (http://www.nyc.gov/html/dcp/html/bytes/dwndistricts.shtml#bcd).

To determine clusters of genetically linked HCV variants among recently and chronically HCV-infected MSM in NYC using phylogenetic analysis, HCV genotypes of recently and chronically HCV-infected individuals were determined based on alignment of Core/E1 polymerase chain reaction (PCR) fragment 432 basepairs. Nucleotide Pairwise Distance (NPD) was performed in Geneious and included reference sequences for subtype 1a and 1b using 21 sequences sampled in NYC from 2001–2005[62, 63]. A phylogenetic tree was created using maximum likelihood estimates to identify clusters of genetically linked HCV variants. Phylogenies were estimated using a maximum likelihood approach and those sequences linked by nodes with bootstrap support >70 considered clustered.

## Results

Of 1,458 men enrolled in the NYC M2M study between 2010 and 2013, 1,030 (71%) consented to serum storage. Excluding two samples with insufficient serum volume for HCV lab analysis, 1,028 men were included. Of these 1,028 men, 79.7% were HIV-negative and 20.3% HIV-positive by testing and self-report (Table 1); 62 (6%) refused HIV testing, of whom 42% (26/62) self-reported being HIV-positive. Of 1,028 men tested, 29/1028 (2.8%) were HCV antibody-positive. Of these 29 who were HCV antibody-positive, 12/29 (41%) were HCV RNA-positive (11 subtype 1a and 1 subtype 1b). Two of 12 HCV RNA-positive participants had low antibody avidity values, suggesting recent HCV infection. Table 1 shows the HCV infection outcomes based on testing, stratified by HIV serostatus by testing and self-report at the NYC M2M study visit.

**Table 1:** HCV infection outcomes based on HIV status, N = 1,028.

HCV Infection Outcome	Overall N	HIV-positive*	HIV-negative/indeteminate*	P-value
		N (%)	N (%)	
Recent	2	2 (100.0)	0 (0.0)	<0.001**
Chronic	10	8 (80.0)	2 (20.0)	
Resolved	17	7 (41.2)	10 (58.8)	
No HCV exposure	999	192 (19.2)	807 (80.7)	
Total	1,028	209 (20.3)	819 (79.7)	

*HIV serostatus based on HIV self-report at study visit

**Chi-square test recent/chronic/resolved and no infection test of association with HIV status

Table 2 compares the sociodemographic and risk behavior characteristics between the men who had HCV antibody seropositivity and those who did not have seropositivity.

**Table 2:** Sociodemographic and risk behavior characteristics, N = 1,028.

	HCV antibody seropositivity*	No HCV antibody seropositivity	P-value	Adjusted OR (aOR)
	(N = 29)	(N = 999)		(95% CI)
Sociodemographics				
Age (years)			<0.0001	
18–24	2 (6.9)	264 (26.4)		-
25–29	5 (17.2)	276 (27.6)		-
30–39	4 (1.8)	238 (23.8)		-
40+	18 (62.1)	221 (22.1)		3.56 (1.57, 8.08)
Race/ethnicity			0.45	-
Hispanic	11 (37.9)	300 (30.1)		
Black Non-Hispanic only	8 (27.6)	231 (23.2)		
White Non-Hispanic only	9 (31.0)	346 (34.7)		
Mixed race or other	1 (3.5)	120 (12.0)		
Education			0.04	-
Less than high school graduate	4 (13.8)	49 (4.9)		
High school graduate	6 (20.7)	113 (11.3)		
Some college	9 (31.0)	325 (32.5)		
College graduate or more	10 (35.5)	512 (51.3)		
Annual household income			0.004	-
< $10,000	10 (37.0)	160 (16.7)		
$10,000 - < $40,000	12 (44.4)	370 (38.6)		
$40,000 - < $60,000	5 (18.5)	254 (26.5)		
≥ $60,000	0 (0.0)	175 (18.3)		
Employment			0.0005	-
Working full-time	9 (31.0)	423 (42.3)		
Working part-time	4 (13.8)	234 (23.4)		
Not working but looking	5 (17.2)	191 (19.1)		
Not working, not actively looking	6 (20.7)	41 (4.1)		
Other	5 (17.3)	110 (11.0)		
HIV serostatus based on HIV testing and self-report			<0.0001	
Positive	17 (58.6)	192 (19.2)		3.18 (1.40, 7.22)
Negative	12 (41.4)	765 (76.6)		-
Indeterminate	0 (0.0)	42 (4.2)		-
Risk behaviors				
General alcohol use in last 3 mos.	22 (75.9)	919 (92.0)	0.008	-
Substance use in last 3 mos.	22 (75.9)	721 (72.2)	0.83	-
Injection drug use ever	8 (27.6)	45 (4.5)	<0.0001	4.34 (1.69, 11.17)
Injection drug use in last 3 mos.	2 (7.1)	5 (0.5)	0.014	-
Alcohol and/or substance use during condomless sex in last 3 mos.**	9 (31.0)	342 (34.2)	0.84	-
Self-reported STIs	8 (27.6)	75 (7.5)	0.001	2.81 (1.11, 7.13)
Median number of sex partners in last 3 mos. (IQR)**				-
Male partners	2 (1, 4)	3 (1, 4)	0.50	
Transgender partners	0 (0, 0)	0 (0, 0)	0.57	
Female partners	0 (0, 0)	0 (0, 0)	0.66	
Any anonymous sex partner in last 3 mos.**	10 (35.5)	313 (31.3)	0.69	-
Any sex partner met on the internet or mobile application in last 3 mos.**	16 (55.2)	623 (62.4)	0.44	-
Any condomless insertive anal intercourse with a male partner in last 3 mos.**	6 (20.7)	141 (14.1)	0.29	-
Any condomless receptive anal intercourse with a male partner in last 3 mos.**	7 (24.1)	166 (16.6)	0.31	-

* HCV antibody seropositivity includes those with recent, chronic, and resolved HCV infections.

** Variables obtained from the sexual network inventory, all other variables obtained from ACASI.

aOR: adjusted odds ratio

95% CI: 95% confidence interval

STIs: sexually transmitted infections

IQR: interquartile range

Those with HCV antibody seropositivity were more likely to be older, have lower education level, have lower annual household income, not work full-time, be HIV-positive, not report recent alcohol use, report IDU recently and ever in the past, and self-report recent STIs.

Based on multivariable logistic regression model, the following variables were significantly associated with HCV antibody seropositivity: age >40 years vs. age ≤ 40 years), adjusted odds ratio (aOR) 3.56 (95% CI 1.57, 8.08); HIV-positive serostatus (vs. HIV-negative/indeterminate status), aOR 3.18 (95% CI 1.40, 7.22), any STI in the last 3 months, aOR 2.81 (95% CI 1.11, 7.13), and IDU ever, aOR 4.34 (95% CI 1.69, 11.17).

**Fig 1:**
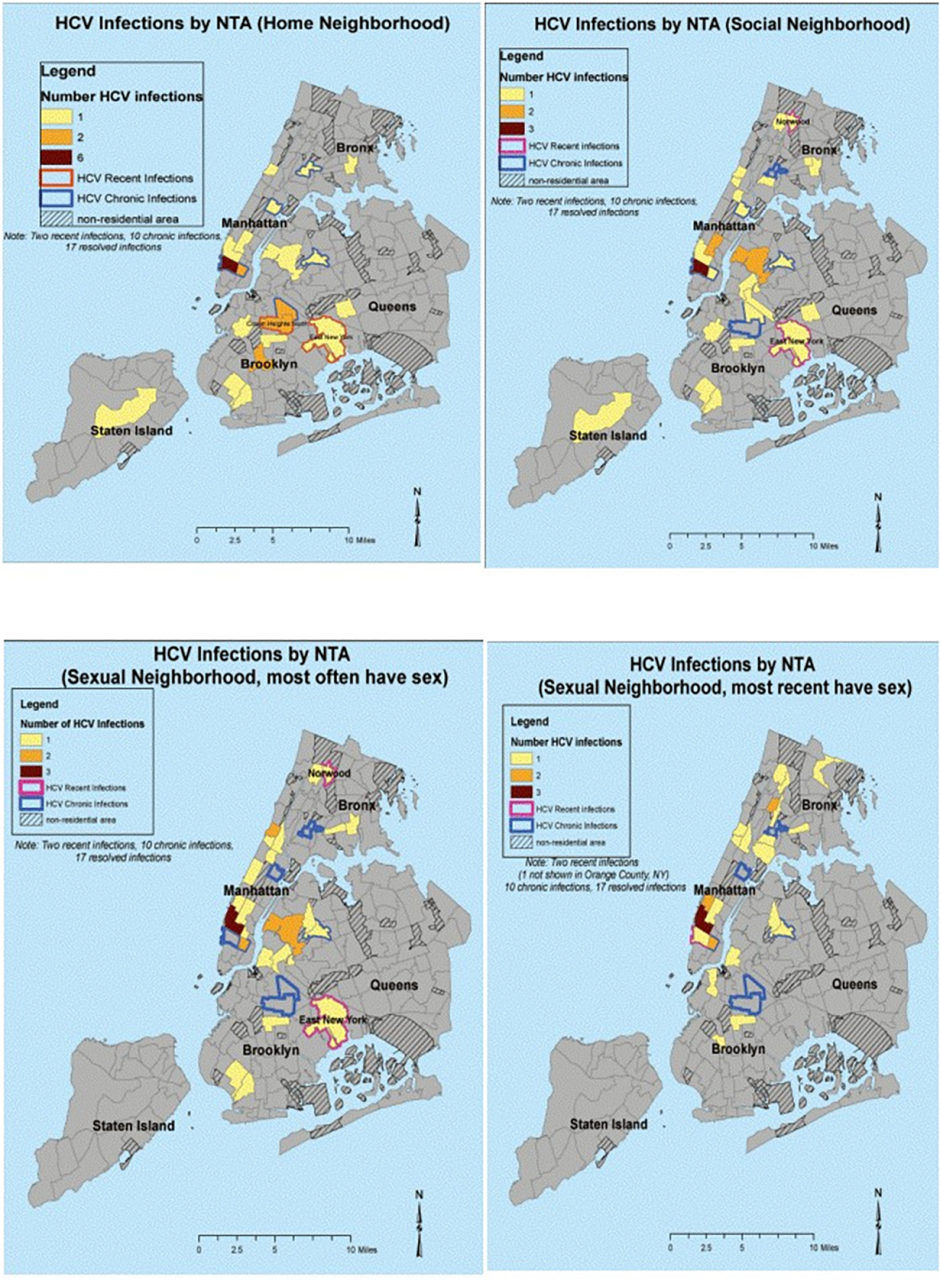
Maps of HCV infections by neighborhoods.

Fig 1 depicts the maps of HCV seroprevalence by neighborhoods at the NTA level, with home (where the men live), social (where the men socialize), and sexual neighborhoods (where they most often have sex in the last 3 months and where they most recently had sex). NTAs in solid colors represent number of HCV seroprevalence as indicated in the legend of each map, while NTAs outlined in red and blue depict recent and chronic HCV infections, respectively. HCV seropositivity was found in all five NYC boroughs, with most concentrated in mid/lower Manhattan for home, social, and sexual neighborhoods. The two recent HCV infections were detected in two different NTAs in Brooklyn for home neighborhoods, with change to Brooklyn, Manhattan, and Bronx NTAs for social and sexual neighborhoods.

**Fig 2:**
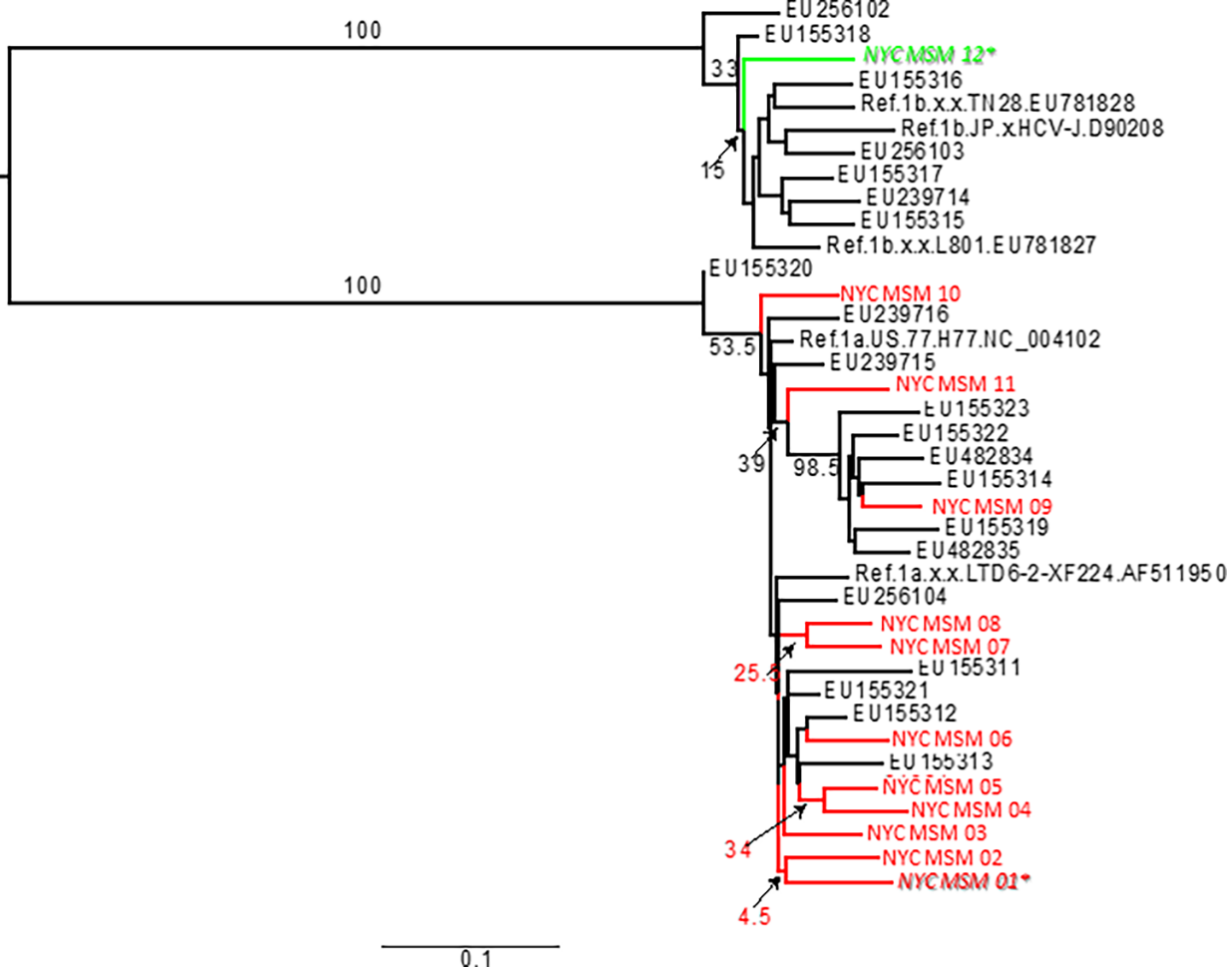
Phylogenetic analysis.

Fig 2 depicts the phylogenetic tree comparing sequence data generated from the 11 NYC M2M participants with subtype 1a HCV infection (labelled “NYC” and in red) and 1 NYC M2M participant with subtype 1b HCV infection (labelled “NYC” and in green), as well as reference sequences for subtype 1a and 1b including 21 sequences sampled in NYC from 2001–2015 from Kuntzen et al[62]. The average nucleotide pairwise distance (NPD) among 11 NYC M2M study subtype 1a was 90.1%; the average NPD among Kuntzen et al. subtype 1a was 89.4%; the average NPD among Kuntzen et al. subtype 1b was 92%. The average NPD among NYC M2M study and Kuntzen et al. subtype 1a was 90.2%, while the average NPD among NYC M2M study subtype 1b and Kuntzen et al. subtype 1b was 89.9%. The two recent HCV infections in the NYC M2M study (denoted with italics) were not clustered together.

## Discussion

This study involving a large sample of community-recruited HIV negative and HIV positive MSM in NYC found an overall HCV seroprevalence of 2.8%. The community-recruited sample had a low prevalence of individuals who reported injecting drugs ever in the past of 5.2%, although they did report sexual risk behaviors that would place them at risk for sexual transmission of HCV, with 14.2% of the MSM reporting any condomless insertive anal sex and 16.8% reporting any condomless receptive anal sex with a male partner in the last 3 months. We found a higher HCV prevalence among HIV-positive MSM compared to HIV-negative MSM (8.1% vs. 1.5%, respectively, p = <0.001), consistent with previously published data showing higher rates of HCV infection among HIV-infected compared to uninfected MSM[13, 15–25]. Of the 29 men who were HCV antibody-positive in our study, only 12 or 41% were HCV RNA-positive, indicating chronic hepatitis C infection. Our HCV seroprevalence of 8.1% (17/209) among HIV-infected MSM is lower than what has been reported in the literature for NYC. In one study, the proportion of HCV infections among HIV-infected individuals in NYC who are MSM dramatically increased from 7% in 2000 to 24% in 2010[64]. In another NYC study from 1999–2007, 10.5% of HCV and HIV co-infected individuals were found to be non-IDU MSM; this study had an older sample of individuals with mean age of 50.4 years[65]. These rates are markedly higher than those reported in other U.S. cities, including San Francisco where HCV prevalence among non-IDU HIV-positive MSM decreased from 9% in 2004 to 2% in 2011[66, 67].

In our study, using HCV antibody avidity testing, we detected recent HCV infection in only two participants, with no evidence of a clustered HCV epidemic based on phylogenetic analysis. This finding differs from a previous finding of an HCV epidemic cluster in NYC, perhaps because our study encompassed a community-recruited sample of mostly HIV-uninfected MSM, with a proportion of HIV-infected MSM, whereas the other study focused solely on HIV-infected MSM who reported no injection drug use. In the NYC study among HIV-infected MSM using phylogenetic analysis, 74 cases of recent sexually acquired HCV infection were reported from 2005–2010, with five clusters of genetically linked HCV strains documented among the men[11]. In this study, condomless receptive anal intercourse and sex while high on methamphetamines were independent risk factors for recent HCV infection. The predominant HCV subtype in our study was subtype 1a, followed by subtype 1b. Several different HCV genotypes have been documented in sexual transmission among MSM. In the NYC study described above, a vast majority (90%) of the sexually-transmitted HCV infections among HIV-infected MSM were genotype 1a, with 6% being genotype 1b[11]. In a Dutch cohort study among HIV-infected and uninfected MSM, most HCV infections were genotype 1, followed by genotype 4[68]. In another Dutch study among MSM attending an STI clinic from 2007–2008, phylogenetic analysis demonstrated at least 4 distinct clusters of HCV infection, suggesting sexual transmission of HCV, with most of the HCV isolates being genotype 1a (71%) and 4d (17%), with the remainder being 3a (8%) and 1b (4%)[16].

In multivariate analyses in our study, we found that HCV antibody seropositivity was significantly associated having reported any STI in the last 3 months, along with older age, IDU ever history, and HIV-positive serostatus. In contrast to other studies, methamphetamine use was not significantly associated with HCV seropositivity. This may be due to a Type II error given the low number of HCV antibody-positive participants found in our study. In a study comparing prevalence of HCV infection among IDU MSM and non-IDU MSM in the Multicenter AIDS Cohort Study, HCV antibody seroprevalence among IDU MSM was 42.9%, significantly higher than 4.0% among non-IDU MSM; in addition, HCV clearance was lower among IDU MSM compared to non-IDU MSM. In both IDU and non-IDU MSM, HIV infection, older age, and Black race were associated with higher HCV antibody seroprevalence. In contrast, our study did not find race/ethnicity to be a significant factor, which may be due to a Type II error given the low number of HCV antibody-positive participants found in our study. In this same study, syphilis infection was associated with HCV seroprevalence among non-IDU MSM only[69]. In the Swiss cohort study, past syphilis infection, along with inconsistent condom use, was associated with a 2-fold increased odds of HCV seroconversion[2]. Although annual HCV testing is recommended for HIV-positive MSM and those entering HIV care in the U.S.[70], universal HCV testing for HIV-negative MSM is not currently recommended[18, 71]. In light of our study findings, we agree with annual testing of HCV infection among HIV-positive MSM, given the higher rates of HCV infection seen among HIV-positive MSM compared to HIV-negative MSM. Rather than universal HCV testing in all HIV-negative MSM, we recommend testing of HCV infection among HIV-negative MSM if they report having a recent STI and past IDU use.

A unique feature of our study was the use of the NLQ to map the geographic distribution of HCV infections among MSM at the level of specific NYC neighborhoods in which the individuals lived, socialized, and had sex. To date, epidemiologic research on the sexually transmitted HCV epidemic among MSM in NYC has been based on health department surveillance and vital statistics data[64] or on referral of suspected HCV infections in HIV-infected MSM from physicians to an referral academic medical center[11]. Public health department and vital statistics data often lack detailed information on where HCV infections are occurring, such as specific NYC neighborhoods, to ascertain whether there is geographic variance in HCV infections within NYC, while referral data are limited by selection bias and by the exclusion of HIV- negative MSM. Although our study did not find evidence of a cluster of recent HCV infections and the geographic distribution of HCV infections differed only slightly based on home, social, and sexual neighborhoods, probably limited by small number of detected HCV infections, we encourage future studies to examine not only home neighborhoods but also social and sexual neighborhoods to help identify NYC areas with the highest HCV prevalence to guide prioritization of HCV screening and prevention interventions. In addition, by combining these studies with phylogenetic analysis to determine clusters of genetically linked HCV infections, unexpected HCV transmission networks not recognized by geospatial mapping and behavioral data might be discovered.

There are limitations to this study. First, the mode of HCV acquisition, either via IDU, sexual, or other means such as via blood product transfusions, could not be confirmed in our participants, as the ACASI did not ask specific questions about HCV infection and mode of acquisition. In addition, the ACASI and SSNQ did not specifically ask about use of sex toys, fisting, or bleeding during sex, which have been reported to be associated with sexual transmission of HCV[10]. Second, our sample size, with a total of 1,028 participants, might have not have been adequate and might have limited a full analysis of the factors associated with HCV serostatus Third, we were not able to determine prevalence of HCV reinfection in our study population; high rates of HCV reinfection have been reported among MSM, especially HIV-infected MSM, in the literature[29–32]. In addition, in those MSM with HCV seropositivity and undetectable HCV viral load, we were not able to differentiate between those with HCV spontaneous clearance vs. successful HCV treatment, since HCV treatment history was not specifically asked during the study. Though the use of a systematic sampling scheme in the main NYC M2M study should minimize selection bias, the participants might not be representative of all MSM living in NYC, with high education level and low past injection drug use history reported in our sample as well as the inclusion criteria of the NYC M2M study specifying that they had to report having anal sex in the last 3 months. In addition, the data might not be generalizable to MSM living in other urban areas in the U.S. Furthermore, self-reported data on the ACASI and data reported to interviewers on the SSNQ might not accurately reflect the actual risk behaviors of the participant and of their partners as a result of socially desirable responding. In addition, in this study we defined recent HCV infection using an HCV antibody avidity index <30% and a positive HCV RNA viral load. This definition is not supported by all studies to date[56], though the implications on our findings are limited given the small number of HCV RNA positive samples found in our study. Lastly, there are limitations inherent to the statistical methodology, with cautious interpretation of findings suggested. The lack of association with variables such as methamphetamine use and race/ethnicity might be due to Type I and II errors resulting from the small number of HCV antibody seropositivity and relatively small sample size.

## Conclusions

In conclusion, in a large community recruited sample of HIV-negative and HIV-positive MSM in NYC with low prevalence of IDU, overall HCV seroprevalence was 2.8%. A higher HCV prevalence was found among HIV-positive MSM compared to HIV-negative MSM. Of the 29 men who were HCV antibody-positive in our study, 12 or 41% were HCV RNA-positive, indicating chronic HCV infection. Only 2 recent HCV infections were detected based on HCV avidity testing, with no clustering of genetically linked HCV infections based on phylogenetic analysis. HCV antibody seropositivity was significantly associated having reported any recent STI, older age, IDU ever history, and HIV-positive serostatus. Our results support testing of HCV infection among HIV-negative MSM if they report having a recent STI and IDU ever in the past rather than universal HCV testing in all HIV-negative MSM.
